# Peripheral Ulcerative Keratitis Associated with Autoimmune Disease: Pathogenesis and Treatment

**DOI:** 10.1155/2017/7298026

**Published:** 2017-07-13

**Authors:** Yan Cao, Wensong Zhang, Jie Wu, Hong Zhang, Hongyan Zhou

**Affiliations:** ^1^Department of Ophthalmology, China-Japan Union Hospital of Jilin University, Changchun City, China; ^2^Department of Ophthalmology, The Second Hospital of Jilin University, Changchun City, China

## Abstract

Peripheral ulcerative keratitis (PUK) is type of crescent-shaped inflammatory damage that occurs in the limbal region of the cornea. PUK is always combined with an epithelial defect and the destruction of the peripheral corneal stroma. PUK may have a connection to systemic conditions, such as long-standing rheumatoid arthritis (RA), systemic lupus erythematosus (SLE), Wegener granulomatosis (WG), relapsing polychondritis, classic polyarteritis nodosa and its variants, microscopic polyangiitis, and Churg-Strauss syndrome. However, the most common connection is with RA, which is also the focus of this review. The pathogenesis of PUK is still unclear. It is thought that circulating immune complexes and cytokines exert an important influence on the progression of this syndrome. Treatment is applied to inhibit certain aspects of PUK pathogenesis.

## 1. Introduction

Autoimmune diseases (AIDs) are systemic inflammatory diseases that generally involve most of the organs in the body, such as the synovium of the diarthrodial joints. Rheumatoid arthritis (RA) and systemic lupus erythematosus (SLE) are the most common AIDs. The aetiology of AIDs is a topic of research, and AIDs lead to many complications. The eye, especially the ocular surface, is frequently invaded by AIDs. In some cases, it may be the first sign of the disease [1]. In addition to corneal damage, which is a rare and late manifestation, other common ocular manifestations are keratoconjunctivitis sicca (KCS), episcleritis, scleritis, uveitis, and retinal vasculitis [2]. The most frequent ocular manifestation of AIDs is dry eye disease (DED, keratoconjunctivitis sicca) [3]. However, the most destructive and severe ophthalmological manifestation of AIDs is peripheral ulcerative keratitis (PUK) [2]. The clinical manifestations of PUK include ocular irritation, pain, redness, photophobia, and corneal opacity. One serious complication of PUK that is associated with RA and other AIDs is corneal perforation and loss of vision [2, 4]. The ulceration may occur in either central or paracentral regions [2]. Due to the peculiarity of the peripheral cornea, it is well vascularized and available for the deposition of circulating immune complexes via the capillary network, and the pathological manifestations are more likely to be present in the periphery. Corneal extracellular stroma is composed of highly organized lamellae of collagen fibrils embedded in a framework of glycosaminoglycans [5]. Fibrocytes and macrophages express human leukocyte antigen DR (HLA-DR) in the corneal matrix, facilitating the direct antigenic stimulation and/or production of local inflammatory mediators [6]. Research suggests that both humoral and cellular immunity are associated with the pathogenesis of autoimmune systemic diseases [7, 8]. A common and recommended therapeutic method for autoimmune disease patients with PUK is systemic medicine, including nonsteroidal anti-inflammatory drugs (NSAIDS), corticosteroids, systemic immunosuppressive chemotherapy [8, 9], and surgical therapy. Recently, biological therapy has become a novel therapeutic method.

## 2. Epidemiology

The epidemiology of PUK associated with autoimmune disease is difficult to investigate preciously, because some autoimmune diseases are common while others are not. According to an investigation, PUK was the second most common ocular complication of autoimmune diseases compared with anterior uveitis, which ranks first place. In a previous study, among the three most frequent underlying diseases, rheumatoid arthritis occupied 34–42% of samples with PUK. SLE and GPA are on its heels [10].

## 3. Rheumatoid Arthritis

RA is a chronic inflammatory and autoimmune disease. The disorder can lead to lacrimal gland destruction and ocular surface inflammation [9]. The characteristic pathological changes associated with RA are pannus formation and synovial hyperplasia, resulting from proliferating fibroblasts and activated immune cells [11].

### 3.1. Pathogenesis

Under normal conditions, the immune system has no response to autologous antigens. The general hypothesis predicts that synoviocytes transform into autoantigens due to a combination of genetic and environmental risk factors. Recently, it has been demonstrated that epigenetic changes are associated with autoimmune diseases. Autoantigens lead to the activation of antigen-specific T and B cells, which eventually contribute to damage in the joints and on the ocular surface [7, 8, 12]. Peripheral corneal ulceration is a devastating complication of RA. Destructive inflammatory cells accumulate in the margin of the corneal stroma and then form a crescent-shaped region at the cornea, causing an epithelial defect, progressive stromal degradation, and thinning, which may lead to perforation and blindness [13]. However, the pathogenesis of PUK associated with RA has not been elucidated. Research suggests that both T cells and antibodies related to RA are involved in the disease [7, 8]. Reputedly, T cells lead to antibody production and the formation of immune complexes that are deposited in the marginal cornea [8, 14]. In addition to other factors, resident antigen-presenting cells, cytokines, and chemokines are counted [15]. The antigen-antibody complexes are more readily available for deposition in the peripheral cornea. The peripheral cornea has abundant materials that are required by AIDs, such as Langerhans' cells, immune globulin, and complement [16].

#### 3.1.1. T Cells in PUK Associated with RA

Antigen presentation to CD4+ T cells is restricted by major histocompatibility complex (MHC) class II [17]. HLA-DR is an MHC class II cell surface receptor expressed by antigen-presenting cells (APCs). HLA-DR, which presents antigens to T cells, is confirmed to play an important role in not only the initiation but also in the maintenance of RA [18].

T cells occupy an important position in autoimmune diseases. Based on the type of T cell receptor (TCR), T cells can be sorted into gamma delta or alpha beta groups. The latter are further classified into CD4+ T cells and CD8+ T cells via the coreceptor molecules CD4 and CD8 expressed on their surface. Unlike CD4+ T cells or T helper (Th) cells, CD8+ T cells are known to destroy/kill cells that have been infected with foreign invading microorganisms. Both CD4+ and CD8+ T cells are important [19]. Nonetheless, the number of CD4+ T cells, but not CD8+ T cells, is significantly higher in patients with RA [20], which indicates that they are potential effectors in RA [21]. CD4+ T cells, when stimulated by cytokines and other effectors, differentiate into diverse T helper cell subsets. CD4+ Th cells can be divided into three different types based on cytokine characteristics: Th1 (which secrete tumour necrosis factor- (TNF-) *γ*), Th2 (which secrete interleukin- (IL-) 4, IL-5, and IL-13), and Th17 (which secrete IL-17) cells [22]. The presence of interferon-gamma (IFN-*γ*) and local APCs that secrete IL-12 is a key to the differentiation of CD4+ T cells into T helper type 1 (Th1) cells. The exposure of CD4+ T cells to IL-4 transforms them into TH2 cells. It is evident that the balance of Th1/Th2 contributes to disease initiation, maintenance, and amelioration [19]. IFN-*γ* derived from Th1 leads to an increased incidence and severity of arthritis and enhances proteoglycan-specific IgG2a antibody responses [23]. The pathogenesis is both Th1 and Th17 mediated [24–26]. Overwhelming evidence indicates that T cells are activated in the peripheral blood and then move to the joints [27]. CC-type chemokines, along with their receptors, help T cells migrate to the joints and other inflammatory sites, where they secrete proinflammatory cytokines that attract and activate monocytes, macrophages, and other cells [17, 27, 28]. Within these sites, activated T cell subsets (e.g., Th1, Th2, and Th17) contribute to the pathology of RA by secreting T lymphokines and stimulating B cell proliferation and autoantibody production [29]. Inflammatory Th1 cytokines, such as IL-1, can be observed in synovial tissue in patients with inflammatory and invasive RA [30]. In particular, IL-17, a proinflammatory cytokine, is strongly associated with the pathogenesis of arthritis [24–26].

The production of Th17 cells is stimulated by a combination of many cell-derived cytokines, such as transforming growth factor-*β*1, IL-6, and IL-21. Moreover, IL-23 is important for the maintenance of IL-17 production. It has been found that IL-1 is also a stimulation factor for IL-17 differentiation in vivo [31–34]. Reportedly, those cytokines are also broadly expressed by epithelial cells [22]. A study conducted by Fossiez and colleagues showed that the addition of IL-17 upregulates the expression of IL-6, IL-8, and granulocyte colony-stimulating factor (G-CSF) [35, 36]. IL-17 results in cartilage destruction and bone erosion via its own function or IL-17-induced TNF-*α*, IL-1*β*, and IL-6 secretion [35]. With the exception of synovial fluid and the T cell-rich area of RA synovial tissue, increased IL-17 is found in both serum and activated peripheral blood mononuclear cells (PBMCs) [35, 37–39]. Compelling evidence has accumulated that tear IL-17 concentration is significantly correlated with ocular surface changes in patients with systemic inflammatory diseases [40]. Furthermore, IL-17 can upregulate B cell proliferation, which suggests that there is an interaction between T and B cells. The differentiation of B cells into plasma cells is also associated with IL-17 [41]. The signature cytokine, interleukin-17 (IL-17), combined with other proinflammatory cytokines (e.g., IL-6, TNF-*α*, IL-1, and IL-8) induced by IL-17 contributes to the promotion of disruptive enzymes such as metalloproteinase-9 [40, 42, 43]. IL-17 induces stromal cells to secrete cytokines, which contribute to inflammatory and haematopoietic processes [36]. IL-17 produced by T helper 17 (Th17) cells and other subsets of T cells, such as gamma delta T cells and natural killer (NK) T cells, can induce the release of certain chemokines, cytokines, and matrix metalloproteinases (MMPs) [44].

#### 3.1.2. B Cells in PUK Associated with RA

The adaptive immune system is characterized by memory, which is not entirely understood [45]. In the beginning, when pathogenic factors invade the human body, naïve B cells migrate to the circulating blood. Naïve B cells then change to activated B cells. There are three different subsequent reactions secondary to the first step. First, with the help of T cells in a germinal centre-like reaction, B cells mature into memory B cells and long-lived plasma cells (LL-PCs). Second, activated B cells grow into short-lived plasma cells (SL-PCs). Third, LL-PCs and SL-PCs derived from activated B cells exist in a common state without T cells [46–48]. Long-term memory and LL-PCs constitute the second line of defence in the immune system [45].

The exact immunopathogenesis of RA is not completely elucidated. However, we have learned from previous experiments that B cell functions, such as the deletion of memory B cells, the interruption of immune activation, the antigen presentation, and the production of inflammatory cytokines, contribute to the progression of pathology [49]. Under normal conditions, B cells have tolerance to self-antigens. However, in RA, this mechanism does not work. Rheumatoid factor (RF), a self-antibody that binds to the Fc portion of human IgG, was shown to be associated with RA in the late 1930s. In the last century, anticitrullinated protein antibodies were discovered. However, anticyclic citrullinated peptide antibodies (anti-CCP antibodies) are more sensitive than RF, and they are special markers of systemic involvement in RA [50]. Compared with RF, anti-CCP appears to be related to increased and worse ocular manifestation in RA [51]. More recently, other posttranslational modifications (PTMs) associated with RA have been found to include remarkable antibodies [45]. Autoantibodies combine with self-antigens to form immune complexes, which lead to the activation of B cells and follicular dendritic cells (FDCs) through receptor systems expressed on the surface of B cells, such as Fc receptors, complement receptors 1 and 2 (CR1 and CR2, also known as CD21 and CD35), and B cell receptors (BCRs) [52, 53].

In addition to producing autoantibodies, B cells can also secrete cytokines in the synovial environment [54]. However, some evidence indicates that B cells can produce diverse cytokines that stimulate pathogenic T cell responses. In the healthy human body, via BCRs and CD40, peripheral blood B cells are a relevant source of IL-6, TNF, and LT in RA. IL-6 has been shown to regulate the balance between effector Th17 cells and Tregs [30]. Th17 and Th17 cell-secreted cytokines have been shown to promote B cell proliferation, differentiation, class-switch recombination, and antibody production in vivo [55]. Moreover, B cells can regulate the Th1/Th2 balance and prevent innate cell-mediated inflammatory responses via the augmentation of IL-10 [56–58].

Furthermore, B cells include a broad spectrum of APCs. Antigens can be processed into antigenic peptides. These antigenic peptides are then presented by B cells via MHC class II molecules. Eventually, T cells will be activated and secrete cytokines associated with RA. Therefore, B cells exert influence on the function of T cells in patients with RA [49].

The abnormal expression of HLA class II antigens on corneal epithelial cells and keratocytes in the area of the ulcer and vasculitis of the adjacent conjunctiva is responsible for the pathology of PUK [59–61]. With the help of immune cell clustering, pannus formation leads to an augmentation in the number of blood vessels in the peripheral cornea via angiogenesis [62]. The vascular architecture of the limbus promotes the deposition of immune complexes in the periphery, which activates the classical pathway of the complement system. The next step is that inflammatory cells, particularly neutrophils and macrophages, accumulate in the peripheral cornea. These inflammatory cells produce collagenases and other proteases, which results in corneal stroma destruction. The release of proinflammatory cytokines, such as interleukin-1, induces stromal keratocytes to produce matrix metalloproteinase-1 and matrix metalloproteinase-2 [63]. Initially, circulating autoantibodies attack specific corneal proteins and initiate the progression of PUK when there is corneal epithelial damage [5].

#### 3.1.3. Matrix Metalloproteinases (MMPs) in Peripheral Ulcerative Keratitis

The corneal extracellular stroma is composed of highly organized lamellae of collagen fibrils embedded in a framework of glycosaminoglycans. There are flattened fibroblasts (keratocytes), macrophages, lymphocytes, and polymorphonuclear leucocytes between adjacent lamellae [5]. MMPs are a type of proteolytic enzyme that degrade specific extracellular matrix components [66]. Depending on the study, local fibroblasts, invading mononuclear cells, and granulocytes are the main cellular source of MMPs associated with RA [6, 59, 65].

**Figure 1:**
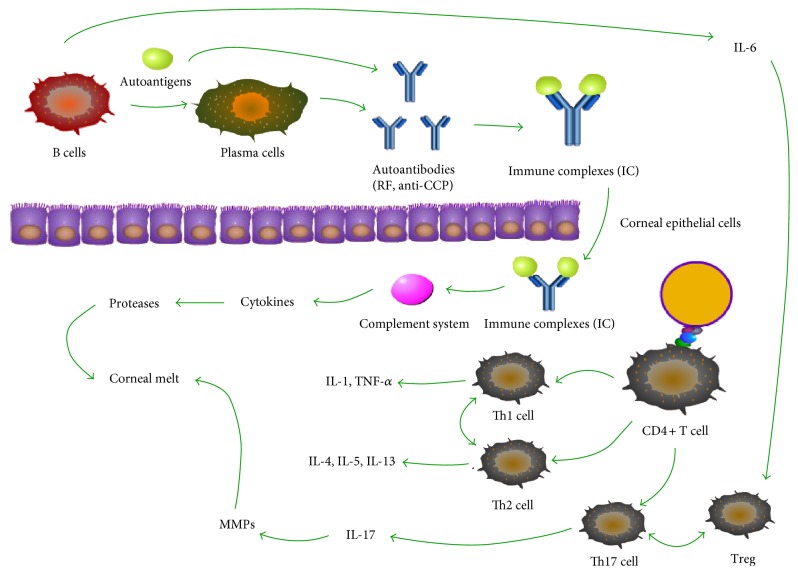
Research suggests that both humoral and cellular immunity are associated with the pathogenesis of autoimmune systemic diseases [7, 8]. Autoantigens lead to the activation of antigen-specific T and B cells. Then, immune complexes and cytokines are formed. It is collagenases and other proteases that eventually contribute to damage in the joints and on the ocular surface [7, 8, 12].

It has been demonstrated that an imbalance between MMPs and their tissue inhibitors (TIMPs) contributes to disease progression [66]. TIMPs are abundant in the abnormal corneal stroma and inhibit metalloproteinase activity and maintain corneal integrity [6]. TIMP, which inhibits collagenase activity and tissue destruction, was deficient when there was a lesion in the cornea [67]. Different subsets of MMPs have similar structures and mechanisms despite their substrate specificities [65, 68, 69]. Previously, MMP-1 has been shown to play a dominant role in dissolving type-1 collagen, which has long been indicated as the pathogenic factor associated with PUK [70, 71]. MMP-1 can clear fibrillar collagens within the collagen triple-helix [6, 64, 72]. Recently, an investigation suggested that MMP-2 secreted by corneal keratocytes and MMP-9 secreted by cells from the lacrimal gland, the conjunctival epithelium, the corneal epithelium itself, or invasive inflammatory cells are both involved in this disease. These enzymes could be produced by invasive inflammatory cells conveyed from the neovascularization located in the limbic cornea [70]. Through a complex subsequent reaction, the collagens will be hydrolysed by the release of collagenases and proteases from lysosomes [66, 70]. MMP-1 is correlated with corneal perforation in patients with PUK. MMP-1 and MMP-8 are necessary for the initiation of the degradation of fibrillar type I collagen, the major component of the corneal stroma. MMP-2 and MMP-9 are enzymes that are needed to hydrolyse type IV collagen, the major component of basement membranes [65]. We hypothesized that these enzymes also limit tissue repair and facilitate the infiltration of inflammatory cells and their proteolytic enzymes (including the MMP that hydrolyses type 1 collagen) into the corneal stroma by breaching the corneal basement membranes (epithelial cells and Descemet's membrane) [70] (Figure 1).

#### 3.1.4. Other Factors of Pathogenesis

Addition to MMPs, a number of apoptotic and proteolytic stromata do a lot in promoting corneal melt. There is a statement that the interaction of epithelial and stromal cells also contributes to destroying cornea structure. Moreover, a drug named nonsteroidal anti-inflammatory drugs (NSAIDs) was proved to cause corneal destruction when they are taken orally [66]. At the last, unknown fields of the pathogenesis are expected to explore.

### 3.2. Treatment

#### 3.2.1. Topical Treatment

In some cases, topical steroids, antibiotics, and even surgical therapy with conjunctival resection of the inflamed area, as well as amniotic membrane grafting, may be successful local treatments for unilateral PUK and systemic-associated disease [74].

The topical usage of corticosteroids can directly target a corneal lesion at the time of application and decrease systemic side effects. After steroid treatment, the defect significantly declines, but epithelial healing is delayed [75, 76].

As an immunosuppressive drug, cyclosporine (cyclosporine A) is the most commonly used agent. Cyclosporine A targets antigen-triggered signal transduction in T lymphocytes and inhibits the expression of many lymphokines (IL-2) and antiapoptotic proteins [77]. The topical application of cyclosporine largely avoids its nephrotoxicity.

Collagenase inhibitors or collagenase synthetase inhibitors function to stop collagenase from destroying the structure of the corneal stroma. Topical l% medroxyprogesterone and topical 20% acetylcysteine have been used in the clinic for several years. Topical corticosteroids should be considered carefully before they are used for patients with PUK related to systemic autoimmune diseases. Although these drugs have immunosuppressive effects, they inhibit new collagen production and thereby increase the risk of perforation [42, 78].

#### 3.2.2. Surgical Treatment

When corneal perforation occurs, procedures that employ cyanoacrylate glue, conjunctival resection of the inflamed area, conjunctival flap, lamellar patch flap, or penetrating keratoplasty may be necessary [72]. Corneal glue is a good alternative and can delay the urgent need for keratoplasty. In some cases, PUK can also affect adjacent tissues, including conjunctiva, episclera, and sclera. Then, the damage of adjacent conjunctiva would promote corneal perforation. Therefore, conjunctival resection of the inflamed area can be used for treatment. Conjunctival flaps can promote healing and put off corneal perforation. Therefore, it is efficient in indolent progression of corneal perforation but not active suppurative keratitis due to the continued leak under the flap. Compared with penetrating keratoplasty, lamellar patch flap is a good choice when there is a high risk of graft rejection. According to the size and location of the corneal perforation, penetrating keratoplasty is required for a large perforation (≥3 mm diameter) [79].

#### 3.2.3. Systemic Treatment

*(1) Corticosteroids*. PUK requires timely diagnosis and treatment. The common and recommended therapeutic method for RA patients with PUK is the use of systemic medicine, including NSAIDS, corticosteroids, and systemic immunosuppressive chemotherapy [80]. Based on various past authoritative tests, ocular surface disorders, especially autoimmune keratitis, significantly benefit from the systemic administration of corticosteroids. In the clinic, ophthalmic surgeons prefer prednisolone because it has the superiority of moderate glucocorticoid effects, mild effects of electrolyte metabolism, and a moderate half-life [75]. However, systemic corticosteroids are inefficient in curing the disorder [80].

*(2) Immunosuppressive Agents*. A study showed that patients who suffer from PUK associated with RA have less mortality and ocular morbidity when they are treated with immunosuppressive medication (cyclophosphamide, methotrexate (MTX), azathioprine, and cyclosporine) [2]. A recent study indicated that MTX combined with cyclophosphamide (CTX) led to Treg malfunction and Th17 suppression by interrupting the maturation and antigen-presenting ability of dendritic cells [81, 82]. MTX (7.5–25 mg/week) and azathioprine (1.0–2.5 mg/kg/day) are the two most appropriate choices if patients are unresponsive to oral corticosteroids and have recalcitrant rheumatoid PUK [45]. MTX, an extensively used agent, has a prominent effect on rapidly proliferating cells (including B cells and T cells) and little effect on resting cells. It can inhibit both humoral and cellular responses [83]. Compared with MTX and azathioprine, mycophenolate mofetil is highly suitable for patients who experience side effects. The oral dose of mycophenolate mofetil is 1.0 g twice daily [45]. In some patients, high doses of systemic corticosteroids and immunosuppressants may fail to control this disease [72, 84, 85].

*(3) Biologic Therapy*. As mentioned above, TNF-*α*, IL-1, and IL-6 play a vital role in rheumatoid keratitis. Recently, biologic therapy has been reported to be effective as the first- or second-line therapy. To date, nine different biologic therapies have already been developed, including seven inhibitors of proinflammatory cytokines (five targeting TNF, one targeting IL-1, and one targeting IL-6), as well as a T- and a B-lymphocyte-targeting agents [86]. Etanercept, infliximab, adalimumab, and golimumab, disease-modifying antirheumatic drugs (DMARDs), have been successfully employed in the treatment of PUK associated with RA. They can inhibit the proinflammatory cytokine TNF-*α* and lead to a decreased production of matrix metalloproteinases, which would halt the progression of corneal stromal lysis [80, 103, 104]. Etanercept is the first TNF inhibitor and was developed in 1998. Etanercept is a recombinant protein that acts as a decoy receptor. It binds to soluble TNF and hampers the combination of TNF and the original TNF receptor. The specific dose of this drug differs from 50 mg once a week to 25 mg twice a week, subcutaneously. It has been demonstrated that the coadministration of etanercept and MTX yields better results [86–89]. Infliximab is the most commonly used TNF-*α*-targeted therapy. It is a chimeric murine/human IgG1 monoclonal antibody that binds to both soluble and transmembrane TNF-*α* [85]. The dosage of infliximab for RA is 3 mg/kg intravenously at weeks 0, 2, and 6 and then every 8 weeks. However, the precise frequency and dosage of infliximab are not clear. Too much or too little of this medicine may lead to the onset of this corneal disorder [72]. Although infliximab has been universally recognized to be a good therapeutic option for PUK associated with systemic immune-mediated conditions, not all patients are sensitive to this drug. By contrast, adalimumab has been demonstrated to be a safe and effective therapy [75]. Golimumab is the same biological drug as infliximab. The drug is administered at 0 and 4 weeks and every 8 weeks thereafter. Golimumab is often used in a hypothetical environment in which MTX alone, as well as other anti-TNF medications, fails to prevent the progression of PUK associated with RA [86, 90–92]. Another inhibitor of TNF-*α* is certolizumab pegol [86]. In addition to TNF-*α*, B cells can contribute to the initiation and maintenance of RA via antigen presentation, autoantibody production, and proinflammatory and anti-inflammatory cytokine secretion. Thus, drugs that target B cells were developed in response to this principle [93]. Rituximab, a monoclonal antibody directed against the CD20 molecule expressed on B cells, can inhibit not only antigen presentation but also antibody/cytokine production. Rituximab contributes to the depletion of sanguimotor B cells. It is an excellent option when at least two DMARDS, including at least one anti-TNF agent, have failed in individuals with RA [83, 94, 95]. The course of treatment begins as soon as 4 weeks after the last dose of etanercept and 8 weeks after the last dose of infliximab or adalimumab. With the previous use of a TNF inhibitor, the dose of rituximab is decreased to 1000 mg per infusion on days 1 and 15 [94]. Other medications include anakinra (a recombinant human IL-1 receptor antagonist), tocilizumab (a humanized anti-human IL-6 receptor antibody of the IgG1 subclass), and abatacept (T cell-blocking Fc portion protein of the extracellular domain of CTLA-4) [86]. IL-6 can regulate the balance between Treg cells and Th17 cells derived from naïve T lymphocytes. It results in a reduced frequency of circulating Th17 cells [82].

IL-17 stimulates PUK associated with RA. Therefore, we need to consider a treatment to suppress this cytokine. Secukinumab and ixekizumab are two humanized monoclonal antibodies that directly block IL-17. Ixekizumab (LY2439821) is a humanized hinge-modified IgG4 IL-17-specific antibody. This drug is in a phase III trial for psoriasis and PsA. Secukinumab (AIN457) is a fully human IL-17-specific IgG1k monoclonal antibody generated by Novartis. This molecule is in phase III trials for chronic plaque psoriasis, PsA, RA, and AS and in phase II trials for chronic noninfectious uveitis. However, the clinical efficacy is not as ideal as expected [96–99]. Brodalumab (AMG827) is a human IL-17-specific antibody developed by Amgen/MedImmune, which was deemed to be efficient in a phase II double-blind, placebo-controlled, dose-ranging study and is currently in a phase II trial for RA and PsA. SCH-900117 and RG4934 are the other two IL-17-targeted antibodies in early clinical development. Ustekinumab (an anti-p40 subunit of the IL-12/IL-23 monoclonal antibody used to cure plaque psoriasis) and guselkumab (a human IL-23-specific monoclonal antibody recently evaluated in psoriasis) are still under investigation [82, 100–102].

## 4. SLE

### 4.1. Pathogenesis

SLE is a chronic and systemic autoimmune disease that affects multiple organ systems, including the joints (arthritis), skin (facial rash, discoid lupus, alopecia, photosensitivity, and Raynaud's phenomenon), kidney (proteinuria), lung (pleuritis), blood (anaemia, leukopenia, and thrombocytopenia), nervous system (psychosis and convulsion), cardiovascular system (pericarditis), and eyes [105]. Various genetic, epigenetic, immunoregulatory, environmental, and infectious factors contribute to the onset and progression of this complex and multifactorial disorder. The inability to clear apoptotic cells may contribute to the initiation of SLE. The destruction of B cell tolerance contributes to ANA production. Both B cells and T cells play an indispensable role in the progression of SLE [106, 109, 111]. In 1967, anti-double-stranded DNA antibodies were found in patients with lupus nephritis. Immune complexes form in this condition, called CRP (which affects apoptotic cell clearance). Apoptotic cells may expose nuclear antigens and allow ANAs to bind extensively. There are two hypotheses to explain tissue damage in SLE. In the former theory, immune complexes are first formed from a combination of anti-double-stranded DNA antibodies and circulating nucleosomes. Second, the complexes are deposited in end-organ capillary beds such as the renal glomerulus. Eventually, tissue damage is caused by inflammatory responses and the activation of the complement system. The latter theory is that these autoantibodies cross-react with normal renal proteins and cause tissue destruction. Internalized self-immune complexes (nucleic acids) that bind to toll-like receptor- (TLR-) 7 and TLR-9 in activated B cells, plasmacytoid dendritic cells, and macrophages start and perpetuate the inflammatory cascade [105, 107, 111]. However, according to statistics, more than 80% of C1q and C4 and over half of C2 homozygous complement-deficient individuals are prone to have SLE.

Tissue injury not only occurs in the location where immune complexes are deposited but also is present at other sites that have been invaded by immune complexes. This may occur because the intravascular activation of complement causes the secretion of C3a and C5a into the circulation, the activation of inflammatory cells, and endothelial damage at regions away from the initial site of immune complex deposition [105]. SLE may affect any structure of the eye (both anterior and posterior segments of the eye, even adnexa). In addition to other ocular manifestations, PUK is a complex and vision-threatening complication of SLE. In the peripheral cornea, immune complexes may deposit in the basement membrane of the endothelial cells. A list of reactions may occur and contribute to corneal melt [108]. There are changes in T cells in patients with SLE, which cause an increase in the proinflammatory Th17 cell population and a decrease in the anti-inflammatory T regulatory cell population [106]. Important steps include T cell activation via antigen binding to the T cell receptor and proper costimulation; T cell activation of B cells; and the production of cytokines such as TNF-*α*, INF-*γ*, IL-10, and a B-lymphocyte stimulator. Th1 cells contribute to the elevated production of IFN-*γ*, which stimulates the dendritic and myeloid cell production of IL-1, IL-6, IL-12, IL-18, TNF-*α*, and B cell survival factor (BAFF), which creates a perpetual proinflammatory cycle [107, 111]. The pathogenesis of PUK associated with SLE is similar to the pathogenesis of PUK in patients with RA in several steps of the reaction, such as Th17, immune complex, and metalloproteinase production.

### 4.2. Treatment

As with RA, the management of SLE also requires drugs and surgical therapy, as mentioned above. The distinction between them may be the frequency and dose. In addition, there are some treatments that are not used for SLE.

IL-2 is responsible for a large proportion of the effect of autoimmune diseases. It acts as a growth, survival, and differentiation factor for activated T cells, as well as a propellant of the differentiation of effector cytolytic T cells and activation-induced cell death (AICD). Therefore, the inhibition of IL-2 is a significant method for SLE treatment and is also under investigation [109].

Biologic agents are used extensively in the treatment of SLE. However, only rituximab and belimumab have been used in clinical practice, and both of them are B cell targeted. There is not much difference between the use of rituximab in RA and SLE. The second mAb, belimumab, has been shown to be appropriate for use in lupus. Belimumab has been demonstrated to target BAFF [110].

In addition to B cell-targeted agents and TNF-*α*, INF (interferon alpha and gamma), TLRs, pDCs, and JAK/STAT inhibitors are used as therapeutic targets [110].

## 5. Wegener's Granulomatosis (WG)

In addition to RA and SLE, WG is a life-threatening systemic granulomatous vasculitis associated with PUK, the aetiology of which is also not clear. Small arteries and veins, the upper and lower respiratory tracts, and the kidneys are often involved. Unlike classic WG, an autoimmune disorder, “limited” WG is a form of the disease that only involves one or two organs, such as the respiratory tract. Various ocular manifestations of WG may indicate the presen`tation of this disease [112–115].

### 5.1. Pathogenesis

WG, along with microscopic polyangiitis (MPA) and Churg-Strauss syndrome (CSS), is associated with antineutrophil cytoplasm antibody- (ANCA-) associated small vessel vasculature (AAV) [118, 121]. The pathophysiologies are in some ways analogous to each other. Autoantibodies and inflammatory cells are responsible for the occurrence of WG. Autoantibody and inflammatory cells are derived from limbal blood vessels of the cornea [113]. The pathogenesis of PUK in WG can be explained by pathogenic B and T lymphocytes and possibly ANCA autoantibodies [114]. The antineutrophil cytoplasmic antibody (cANCA) test is a specific and sensitive marker in the diagnosis of WG associated with PUK [115]. ANCA binds to cytokine-primed neutrophils and monocytes that express target antigens. Then, the complex contributes to a release of lytic enzymes and proinflammatory cytokines such as IL-8. Moreover, the combination of ANCA and neutrophils leads to endothelial adhesion and cytotoxicity in cultured endothelial cells [118]. The complement pathway, combined with T cells, antigen-specific Th17 cells, and the cytokines IL-17 and IL-23, also plays an important role in the pathogenesis [73, 118–127]. HLA-DR antigens, expressed on vascular endothelial cells, increase the transendothelial migration of CD4+ memory T cells. NK cells have a similar function as CD4+ effector memory T cells. Both of them can secrete cytolytic molecules, perforin, and granzymes [119]. T cell help is needed for the production of ANCA [118]. In addition, Th17 cells that secrete IL-17 were demonstrated to be critical mediators of PUK associated with WG. The presentations of IL-1, IL-6, IL-17, IL-23, TNF-*γ*, and other related cytokines have similar functions in patients with WG and RA.

### 5.2. Treatment

#### 5.2.1. Topical Treatment

Patients who suffer from WG combined with PUK are not sensitive to local corticosteroid therapy unless a subsequent systemic immunosuppressive therapy is used [114].

#### 5.2.2. Surgical Treatment

Traditional therapy may fail to heal intractable WG. According to a study by Lu et at., the combination of conjunctivectomy with cryotherapy is effective. The main theory is that necrotic tissue, immune complexes, inflammatory cells, and protein lysozymes are affected [113]. Corneal perforation is a serious complication of PUK in WG. Surgical reinforcement is necessary for treatment [115].

#### 5.2.3. Systemic Treatment

Both oral and intravenous cyclophosphamide, in combination with corticosteroids, are good choices for patients with this disease. Cyclophosphamide 2 mg/kg per day and prednisone 1 mg/kg per day have been shown to be appropriate for the treatment of PUK with WG by Watkins et al. [116]. Azathioprine is safer and less effective than cyclophosphamide. Limited WG usually responds to MTX, while active ANCA-associated vasculitis responds to rituximab [114]. Abatacept, which targets CTLA4-Ig, is used on a small scale in patients and has good prospects. Campath-1H® (anti-CD52) can reduce circulating lymphocytes and is used in patients with refractory AAV (30 or 60 mg). Inhibitors of IL-17, the IL-17 receptor, IL-12/IL-23, and the C5a receptor are still under investigation [118]. Biological therapy, such as TNF inhibitors, has also not undergone large-scale investigations [117] (Table 1).

## 6. Differential Diagnosis

Patients suffering from peripheral keratitis and ulceration must have a detailed personal and family history about autoimmune systemic diseases. Therefore, collecting medical history is an important part of differential diagnosis [62]. PUK usually occurs in patients with long-standing rheumatoid arthritis. We can learn from a case that there was an average of 19.6 years between diagnosis of RA and PUK. While in WG and other systemic vasculitis, PUK and corneal melt appear early. The visual loss may follow within few days [8]. The different parts of PUK associated autoimmune diseases may be rare. However, we mainly distinguish them by differences of systemic diseases (such as RA, SLE, and WG). That is, symptoms and signs, biomarkers, and other auxiliary examinations are used as the diagnosis basis.

## 7. Conclusions

The specific pathogenesis of PUK associated with autoimmune diseases still remains a mystery. RA, SLE, and AAV are topical disorders of autoimmune diseases and have close correlations with ocular manifestations. The existing data indicate that both humoral immunity and cell-mediated immunity are involved in these ocular effects, which are commonplace among these disorders. However, RA emphasizes the function of CD4+ T cells, while SLE and AAV involve autoantibodies. Proteinases eventually lead to corneal melt. The ocular complications may threaten vision and require timely diagnosis and treatment. Recently, some research has suggested that IL-17 and other drug targets affect people's vision. Traditional therapy, steroids, and immunosuppressive therapies are no longer the issues that scientists focus on. Biological therapy, including the inhibition of B cells (rituximab), T cells (abatacept), IL-1 (anakinra), IL-6 (tocilizumab), and TNF-*α* (etanercept, infliximab, adalimumab, and golimumab), has already been the subject of research and has been incorporated into clinical applications. In the future, with a clearer understanding of the pathogenesis of this disease, the inhibition of IL-17 and other cytokines may result in successful treatment. PUK associated with autoimmune disease will not influence quality of life.

## Figures and Tables

**Table 1:** The therapy of PUK associated with autoimmune diseases.

Therapy	Classification	Agent	Target
Topical therapy	Corticosteroid	Prednisolone	Immune system [74, 75]
	Immunosuppressant	Cyclosporine A	Antigen-triggered signal transduction in T lymphocytes and expression of many lymphokines (IL-2) and antiapoptotic proteins [76]
	Collagenase inhibitors	l% medroxyprogesterone	Collagenase [41, 77]
		20% acetylcysteine	

Surgical therapy	Cyanoacrylate glue, conjunctival flap, lamellar patch flap, or penetrating keratoplasty [71], conjunctivectomy combined with cryotherapy (mentioned in SLE) [111]		

Systemic therapy	Corticosteroid	Prednisolone	Immune system [74, 75]
	Immunosuppressant	MTX	Immune system [2]
		CTX	
		Cyclosporine	
		Azathioprine	
	Biologic therapy	Etanercept	TNF-*α* [80]
		Infliximab	
		Adalimumab	
		Golimumab	
		DMARDs	
		Rituximab	B cell [81, 84, 91–93]
		Etanercept	
		Adalimumab	
		Abatacept	T cell [84]
		Anakinra	IL-1 [84]
		Tocilizumab	IL-6 [84]
		Ixekizumab	IL-17 [94–97]
		Secukinumab	
		Brodalumab	
		Ustekinumab	IL-12/IL-23 [80, 98–100]
		Guselkumab	IL-23 [80, 98–100]
		Belimumab (mentioned in SLE)	BAFF [108]
		INF (interferon alpha and gamma), TLRs, pDCs, and JAK/STAT inhibitors (mentioned in SLE)	INF (interferon alpha and gamma), TLRs, pDCs, and JAK/STAT

Most of the therapies are similar. However, there are some different places among these diseases.
